# Pre-procedural Antimicrobial Mouth Rinse: A Concise Review

**DOI:** 10.7759/cureus.30629

**Published:** 2022-10-24

**Authors:** Safiya Hassan, Prasad Dhadse, Pavan Bajaj, Kiran Sethiya, Chitrika Subhadarsanee

**Affiliations:** 1 Department of Periodontology and Implantology, Sharad Pawar Dental College and Hospital, Datta Meghe Institute of Medical Sciences, Wardha, IND

**Keywords:** antibacterial, chlorhexidine, oral condition, microorganisms, mouthwash

## Abstract

Mouth rinses are therapeutic solutions utilized for gargling and rinsing the oral cavity. Several oral disorders need a mouth rinse, which can differ from halitosis to diseases of the periodontium. It is essential for the management of secondary infections, for example, oral mucositis. A mouth rinse may be suggested as an anti-inflammatory, antibacterial agent, topical analgesic, or for caries prevention. Several different mouth rinses are accessible nowadays. Selection of a suitable mouth rinse depends on the patient’s requirement, disease threat and competence, and safety of mouth rinse. The application of antiseptics to the skin or mucous membranes before surgery or injections has been practised for many years. The goal of such an application is to reduce the number of microorganisms on the surface to prevent their entry into underlying tissues, which could cause bacteremia, septicemia, or harmful local infections. A similar idea of minimizing oral bacteria underlies the patient's usage of an antibiotic mouthwash before dental treatments. This decrease in microflora also reduces the possibility of pathogens entering the patient's mouth through direct contact, splatter, or aerosols during dental treatment. The main aim of this review is to aid oral health care professionals in making the correct selection of mouthwash while dealing with different conditions of the oral cavity.

## Introduction and background

Clinicians, healthcare workers, and patients potentially come in contact with bio-aerosols. These originate from various sources, and every patient is at a higher risk of infection, irrespective of any comorbidity. The potentially pathogenic nature of bioaerosols possesses severe biohazard. Aerosols (solid or liquid particles) with a diameter of less than 50 µm persist in the atmosphere for a very long time. Spatter >50 µm in diameter that is excessively large becomes suspended for longer times in the air [1]. Dental procedures that use three-way syringes, ultrasonic scalers, slow- and high-speed handpieces, and other tools can cause aerosol and splatter. Evidence suggests that aerosol in a dental clinic can spread 1-3 meters from its source and cause infection on distant surfaces. On the contrary, spatter spreads over shorter distances and settles down rapidly. Regarding airborne illness, aerosol in dental clinics is a significant concern [2].

The size of the droplet reduces as it begins to evaporate; it has the potential to remain in the air or return to it as a dust particle. As a result, spatter droplets could also be a possible source of infection in dental treatment practice. Other diseases, including SARS, measles, and herpes viruses, have also been linked to splatter and droplet nuclei transfer. Saliva naturally moistens the oral environment. The saliva and other oral secretions are severely infected with germs and viruses. These organisms are commonly found in dental plaque, both supragingival and the periodontal pocket. The mouth hosts germs and viruses from the nose, throat, and respiratory system as part of this complex. These might include harmful bacteria and viruses in saliva and other oral secretions. Any dental operation that could aerosolize saliva contaminates the air with germs from some or all of these sources [2].

Cross-infection in the dental office can be caused by aerosols released during dental procedures, including commonly occurring oral microorganisms like Streptococcus pyogenes, Veillonella parvula, Actinomyces species, and Fusobacterium nucleatum, pathogenic organisms like Mycobacterium tuberculosis, Legionella pneumophilia, and Staphylococcus species, as well as contagious viruses like HIV, hepatitis B virus, hepatitis C virus, herpes simplex virus, etc. The use of mouth rinses in the oral cavity effectively decreases bacterial counts. Preprocedural mouthwash has been demonstrated to reduce the number of microorganisms in the dental aerosol, lowering the risk of infection in the dental clinic [3].

## Review

Chemical plaque control agents

There are three generations of antiplaque agents in which first-generation antiplaque agents eliminate 20-50% of plaque, and mouth retention is minimal, e.g., quarternary ammonium compounds, phenol, antibiotics, and sanguinarine. In the second generation, the plaque reduction is around 70-90% and is better maintained than in the previous compounds. It has slow-release qualities and improved oral tissue preservation, e.g., bisbiguanides (chlorhexidine). Third-generation antiplaque agents prevent bacteria from adhering to or opposing the tooth. They have a lower retention capacity than second-generation, e.g., delmopinol [4].

Chlorhexidine

Chlorhexidine (CHX) is described as a symmetrical bisbiguanide in its natural state. Its structure consists of two biguanide groups, four chlorophenyl rings, and a hexamethylene bridge. Since CHX is dicationic, its direct effects are seen on safety and efficacy. It is accessible in three types: digluconate, acetate, and hydrochloride salts. CHX is a broad spectrum in nature and has a wide range of antibacterial properties, which are effective against gram-positive and gram-negative bacteria, including anaerobes and aerobes, lipid-enveloped viruses, fungi, and yeasts [5].

CHX enhances membrane permeability along with cellular macromolecule coagulation. It furthermore reduces the Porphyromonas gingivalis attachment to epithelial cells. It doesn’t show any interaction with bacterial receptors or enzymes and therefore doesn’t show resistance from microorganisms. Substantivity is the potency of drugs to bind with hard and soft tissues or adsorb onto them. Its mechanism is so that even after using it once, its actions are seen for a prolonged time and are satisfactory for plaque control. It is determined by pH, concentration, time, and temperature when the solution is kept in the mouth. CHX has a substantivity of eight to twelve hours [6].

Mechanism of action

CHX has three mechanisms of action (MOAs), including cell membrane disruption. CHX binds to the negatively charged sites in a bacterial cell wall due to its cationic nature and impedes osmosis, disrupting the cell wall. CHX gets attached to the desquamated epithelial cells and salivary proteins by blocking acidic groups of salivary glycoprotein, which reduces glycoprotein absorption on the tooth surface and prevents pellicle formation. At low and high concentrations, CHX is bacteriostatic and bactericidal, respectively. CHX depicts prolonged antiseptic release, including bacteriostatic action lasting more than 12 hours. It prevents the absorption of the bacterial cell wall on the tooth surface and prevents plaque formation. It inhibits the maturation of plaque [4]. The dicationic chlorhexidine molecule attaches to the tooth surfaces with one cation and to the bacteria seeking to colonize the tooth surface with the other, known as the Pin-Cushion effect. The Pin-Cushion phenomenon prolongs the impact of CHX. Bacteriostatic activity in the oral cavity lasts approximately 12 hours following a single rinse [2].

Safety

CHX undergoes minimal metabolic cleavage in the body and is absorbed poorly in the gastrointestinal tract (GIT). Thus, it manifests a level of toxicity at a meagre percentage. Its IV lethal dose (LD) is 22mg/kg and oral LD is 1800mg/kg. As revealed by animal studies, the primary route of CHX excretion happens through faeces. The formation of carcinogenic substances is not evident, and no teratogenicity has been seen with its prolonged use. Clinical usage of numerous solutions for CHX mouth rinses is accessible throughout the world. The CHX mouth rinse comprising CHX (0.12%) delivers 18mg of CHX when used at 15ml volume per rinse, and those solutions consisting of CHX (0.2%) deliver 20mg of the total dose of CHX when used at 10ml volume per rinse. The authors concluded that the effectiveness of CHX is dose-dependent and concentration-dependent. Thus, both of these preparations give good results. But CHX with lesser concentration decreases its after-effects while continuing its benefits. To ensure excellent and efficient patient compliance, the recognized duration of rinsing time is 30 seconds. Patients should be directed to mouth rinse after breakfast and before bed, with no less than 30 minutes time intervals after brushing of teeth [7].

Several local adverse effects of CHX mouth rinse include brownish staining of the tongue, restorative materials, and the teeth. It also alters the sensation of taste, mainly for salty taste. The use of a high concentration of CHX rinse leads to mucosal erosion. Parotid swelling can be seen in rare cases, and the amount of supragingival calculus deposition is increased. Several mechanisms for CHX discolouration were proposed [8], including the release of parachloraniline by degradation of CHX (Maillard reactions), non-enzymatic type of browning reactions, metal sulfide formation with the help of protein denaturation by CHX, and cationic antiseptics precipitate anionic dietary chromogens. CHX formulations include mouthwash, gels, sprays, toothpaste, varnishes, sugar-free chewing gum, and periochip.

Listerine mouthwash

The phenolic agents have been in clinical use for the longest time. Joseph Lister first included them in a carbolic spray mixture for surgical antisepsis in 1865. Listerine is probably the oldest oral rinse and contains phenolic compounds, a mix of phenol like eucalyptol, essential oils, and thymol, added with methyl salicylate in a 26.9% hydroalcoholic vehicle and menthol. Clinical studies published in the Journal of American Dental Association (JADA), Volume 125, August 1994, were of short duration, ranging from 7-60 days, and showed statistically significant reductions (around 35%) in the degree of gingivitis and plaque when the mouth rinse utilized with, or in the absence of oral health procedures. Recent studies followed the American Dental Association (ADA) rules, which are six months long. Plaque reduction varied from 20 to 34% after Listerine rinse was used twice daily after brushing teeth, and gingivitis reduction ranged from 28 to 34%. Bacteriologic studies stated that there were no significant alterations in the equilibrium of the oral flora of both the groups, and there was no emergence of presumptive pathogens, potential or opportunistic, in compliance with the ADA guidelines when control and Listerine groups were compared [9]. Comparative studies of Listerine antiseptic and other antiseptic rinses by several investigators have produced contradictory quantitative outcomes. Still, significant reductions in gingivitis and plaque were seen with Listerine [10-12]. Traditionally, the mechanism of Listerine is thought to be cell wall disruption and bacterial enzyme inhibition. Evidence suggests that Listerine can also extract endotoxin from the lipopolysaccharide of gram-negative bacteria, prostaglandin synthetase inhibitory action, and anti-inflammatory properties that can take place at a concentration lower than for antimicrobial activity. Few patients experience an unpleasant taste and an initial burning sensation. Rarely minimal tooth discolouration has been seen, but most clinical studies do not report this problem. Recent research from the National Cancer Institute (NCI) has raised concerns about the connection between mouthwashes with high alcohol concentration and pharyngeal and oral malignancies [13]. Thus NCI did not advise changing their mouth rinse use depending on this retrospective study. Cool Mint Listerine has recently been introduced as a modified product with a lower alcohol content (22%) and a different flavour. The ADA has recognized the improved product.

Plax

Plax (sodium benzoate) is an antibacterial mouth rinse consisting of alcohol 7.5 % and should be used before brushing or alone. Short-term studies indicated a significant decrease in plaque scores with Plax. Various longer-duration studies followed these. A six-month trial and negative studies with single-use protocols found that Plax is no more productive than a placebo mouth rinse. Recent additional studies support this disparity, several studies reported a reduction in plaque, yet another study in a larger group found no significant variance between rinse and placebo [14]. ADA disapproved of this mouthwash [15].

Delmopinol and herbal extract sanguinarine

Delmopinol is derived from morpholinoethanol and has been shown to prevent gingivitis and plaque. The short-term numbness of the tongue, teeth and tongue stains, taste disturbances, and infrequent mucosal erosion and pain are among the adverse effects [16]. Sanguinarine is an anti-plaque/antigingivitis agent presently used in toothpaste and mouth rinses. Sanguinaria canadensis is a bloodroot plant, and it is an alkaloid extract. To enhance the antiplaque effect, the current composition contains 0.03% mixed extract (equal to 0.01% pure sanguinarine) and zinc chloride (0.2%). Some researchers describe a significant decrease in gingivitis and plaque, and others report minimal effect; hence, the rinse's outcome is unclear [10]. A trial using the dentifrice showed that orthodontic patients had significantly less plaque and gingivitis. In other studies, the variations among experimental and control groups were insignificant. No other adverse effects (apart from an infrequent burning sensation) were present in the research, and no microbiologic signs of excessive opportunistic growth of oral microbes [17].

Benzydamine hydrochloride

Antimicrobial, anaesthetic, analgesic, and anti-inflammatory properties are shown by benzydamine hydrochloride. As for the mechanism of action, It decreases pro-inflammatory cytokine production and most likely affects thromboxane and prostaglandin production. According to studies, benzydamine significantly reduces radiation-induced mucositis extent, prevalence, and severity. Hence, it is recommended for ulcerative lesions like recurrent aphthous stomatitis and radiation-induced mucositis [18].

Cetylpyridinium chloride and sodium benzoate

The plaque-inhibitory effect of cetylpyridinium chloride (CPC), a quaternary ammonium molecule, is moderate. As for the mechanism of action, due to its cationic nature, it binds to the membrane of bacterial cells, therefore, causing seepage of intracellular constituents and disruption of the cell membrane. Mucosal retention is provided by the single cationic group, which binds to the mucosa, leaving limited unattached areas for its antimicrobial activity. Sodium benzoate weakens plaque attachment by dispersing fat, carbohydrate, and protein, and it can be easily detached with the help of brushing teeth [19].

Triclosan

Non-ionic antiseptic triclosan (2, 4, 4'-trichloro-2'-hydroxy diphenyl ether) is an anti-inflammatory substance. Numerous studies have revealed that using sodium lauryl sulfate and triclosan lessens the inflammatory reaction on the gingiva and reduces the healing period and severity of recurrent aphthous ulcers. It inhibits lipoxygenase and cyclooxygenase pathways and reduces inflammatory mediators (leukotrienes and prostaglandins). Triclosan also enhances the binding capacity of mouth rinses to the mucosa of the oral cavity and, therefore, is obtainable for a more extended period [20].

Oxygenating agents and povidone-iodine-containing mouthwashes

These are bleaching agents with vigorous oxidizing activity. These antimicrobial agents have a broad spectrum of action. Sodium peroxyborate, polycarbonate, and hydrogen peroxide are oxygenating agents that release nascent oxygen, remove stains, unbind debris, and kill anaerobic bacteria. Mouthwashes containing oxygenating agents are advised for acute ulcerative disorders, to mitigate tenderness produced by orthodontic appliances and dentures, and to remove stains [21]. Povidone-iodine is a wide-spectrum agent with an affinity for bacteria, fungi, viruses, and protozoa. It is an iodophor where povidone is weakly linked to iodine, supplying free iodine to the microbial cell membrane. It lowers the formation of plaque, radiation mucositis, and gingivitis severity. It is contraindicated for people sensitive to iodine or with existing thyroid conditions [22].

Antibacterial peroxidase mouthwashes and fluoride-containing mouthwashes

It consists of enzymes such as lactoperoxidase, glucose oxidase, lactoferrin, and lysozyme, which have an affinity against microbial peroxide. They reinstate the antimicrobial action of saliva, therefore, beneficial in treating gingival inflammation, dry mouth, and oral malodor. Due to low pH, it might cause dental erosion in its long-term use [23]. These mouthwashes contain fluoride in different formulae, such as sodium fluoride (NaF) or acidulated phosphate fluoride (APF). Fluor-hydroxyapatite and fluorapatite aid in the remineralization of enamel, which protects it against acid secretion. As a result, they are helpful for patients with a high risk of developing dental cavities, undergoing orthodontic treatment, and having xerostomia due to radiation therapy. Due to the significant danger of fluoride intake, they are not advised for children under six years of age [24].

Propolis-based mouthwashes

Propolis, a naturally occurring resinous substance produced by honey bees, has recently been proposed as an alternative to anti-plaque rinse. It is most likely due to propolis flavonoid content. Antibacterial activities of propolis have been observed against various strains of the anaerobic and aerobic oral bacterium. It is a highly inflammatory and microbial-resistant mechanism of action: by inhibiting cyclooxygenase and lipoxygenase enzymes. Propolis inhibits prostaglandin production, resulting in an immediate and potent reduction in tissue inflammation and pain. The propolis chemical composition comprises resin (50%), wax (30%), aromatic and essential oils (10%), pollen (5%), and other constituents (5%) [25].

Chlorine dioxide mouthwashes

Chlorine dioxide (ClO2) destroys microbiota by oxygenating and neutralizing the toxins created by oral cavity microorganisms. By assisting in the breakdown of volatile sulfide compounds (VSCs), stabilized ClO2-based products aid in delaying the onset of gingival inflammation. They have microbicidal activity against various oral pathogens. It is a non-alcoholic formulation with no colour or dye. Oxyfresh ClO2 power rinse composition includes deionized water, sodium citrate, zinc acetate, chlorine dioxide concentrate (15% solution), sucralose, xylitol, aloe powder, citric acid, and sodium hydroxide [26].

Effective dental plaque control is essential for preserving periodontal health. Plaque control can be accomplished by combining strict at-home oral hygiene routines with regular dental checkups, during which the dentist or hygienist performs the essential scaling, polishing, and root debridement procedures using ultrasonic scalers and air polishers. It is crucial for people with periodontitis to keep their planned dental appointments, whether in the active or maintenance phases of periodontal therapy. Periodontitis patients must receive ongoing periodontal care promptly to prevent the disease's progression and reinfection, especially in high-risk individuals. Given the bidirectional relationship between periodontal disease and common non-communicable diseases like diabetes mellitus, rheumatoid arthritis, hypertension, and those who are immune suppressed, the progression and reinfection of periodontal pockets affect oral and systemic health. Pre-procedural mouth rinse is one of the most frequently recommended strategies to lower the level of contaminants in the aerosol during dental treatments.

Table 1 summarises articles based on SARS-CoV-2 and aerosol reduction.

**Table 1:** Summary of articles (SARS-CoV-2) Povidone iodine (PVP-I); Hydrogen peroxide (HP); Cetylpyridinium chloride (CPC); Chlorhexidiene (CHX)

Study	Material and Methods	Conclusions
Carrouel et al. 2020 [27]	The test group was rinsed with 30 ml of 0.1% beta-cyclodextrin and citrox mouthwash for 1 min. The Control group was rinsed with distilled water for 1 min. Both the groups used mouth rinse thrice daily.	Authors concluded that mouth rinses and local nasal applications that contain beta-cyclodextrins in combination with flavonoids agents, such as citrox, could be a useful adjunctive treatment to reduce the viral load of saliva and nasopharyngeal microbiota, including potential SARS-CoV-2 virus, if chlorhexidine, a common ingredient of mouthwash, is ineffective at killing SARS-CoV-2.
Huang et al. (2021) [28]	Group 1 administered 0.12% CHX for 30 seconds two times daily and 0.12% CHX in combination with oropharyngeal sprays of 1.5 mL 3 times daily while group 2 was untreated.	62.1% of patients who utilized CHX as an oral rinse had SARS-CoV-2 removed from their oropharynx, compared to 5.5% of the control group. In the combination group, 86.0% of oropharyngeal SARS-CoV-2 was eradicated as opposed to 6.3% of patients.
Costa et al. (2021) [29]	In the test group, 15ml of CHX (0.12%) was administered, and in the control group, an inactive substance was used.	At 5 and 60 minutes after rinsing, the salivary load was much lower than in the control group. In 72% of the volunteers who used chlorhexidine compared to 30% in the control group, there was a decrease in the load of SARS-CoV-2.
Ferrer et al. (2021) [30]	In group 1 PVP-I (2%) , HP (1%), CPC (0.07%), CHX (0.12%)/1 min. In group 2, distilled water	There was no statistically significant difference in the salivary viral load among the mouthwashes evaluated.

As CHX is a well-known antibacterial agent used in dentistry, as seen in Table 2, most of these investigations used it as the primary antiseptic agent to decrease aerosol contamination.

**Table 2:** Table summarizing studies Cetylpyridinium chloride (CPC); Chlorhexidine (CHX); Colony forming units (CFUs); Probing depth (PD); Chlorine dioxide (CLO2); Analysis of variance (ANOVA)

STUDY	COUNTRY	TYPE OF PARTICIPANTS	INTERVENTION (NO.)	NUMBER OF CFU COMPARED WITH CONTROL	AUTHORS’ CONCLUSIONS
Feres et al., 2010 [31]	Brazil	20 natural teeth, <10% with detectable supragingival calculus, <30% with clinical attachment loss and pocket depth 5 millimetres	CPC (0.05%), CHX (0.12%), Water, No rinse	CHX v/s no-rinse: Reduction of 78%, CPC v/s no-rinse: Reduction of 77%, CHX v/s water: Reduction of 70%, CPC v/s water: Reduction of 68%	Mouth rinses comprising CHX (0.12%) and CPC (0.05%) are equally effective in decreasing the amount of spatter microorganisms produced throughout ultrasonic scaling.
Reddy et al., 2012 [32]	India	Systemically healthy subjects	Sterilized Water, Non-tempered CHX (0.2%), Tempered CHX (0.2%)	CHX v/s water: decrease of 1.972 CFU, Tempered CHX v/s water: decrease of 1.984 CFU	Pre-procedural mouth rinse can considerably decrease the live bacterial content of aerosols in dental clinic, and tempered CHX rinse was more effective than non-tempered CHX rinse
Shetty et al., 2013 [33]	India	Minimum of 20 permanent teeth, Plaque index from (1-3), Oral hygiene score from (1.3-3)	Sterile water, CHX (0.2%), Tea tree oil	CHX v.s water: 93.3% decrease	All antiseptic rinses reduce microbial CFU in the samples of aerosol. CHX rinses were found superior to other rinses.
Gupta et al., 2014 [34]	India	Mean plaque score: (2-3) on the plaque index and periodontitis ( 4 sites with PD: 4 mm)	CHX (0.2%), Herbal rinse, Water	CHX v/s water: decrease in 72.05%, Herbal rinse v/s water: decrease in 35.86%	Herbal rinse has shown a good outcome in reducing aerosol infection produced due to ultrasonic scaling, however less effective than CHX rinse (0.2%).
Dawson et al., 2016 [35]	United Kingdom	Patients underwent complete fixed orthodontic therapy and planned for debonding of the fixed appliances.	No rinse, CHX (0.2%)	CXH v/s no-rinse: increase of 77%, CHX v/s water: increase of 25.3%	The usage of pre-procedural water or CHX mouthwash occurred to cause a decrease in the amount and diversity of airborne microorganisms.
Retamal-Valdez et al., 2017 [36]	Brazil	Approx. 80% sites, visible supragingival plaque <10% sites, visible supragingival calculus less than 30% of sites, PD: 5 mm	CPC (0.075%), zinc lactate (0.28%), sodium fluoride (0.05%), CHX (0.12%), Water, No rinse	CXH v/s no rinse: Reduction of 77%, CPC v/s no rinse: Reduction of 70%, CHX v/s water: Reduction of 70%, CPC v/s water: Reduction of 61%	Preprocedural mouthwash consisting of CPC (0.075%), zinc lactate (0.28%), and sodium fluoride (0.05%) was efficacious in decreasing the bacterial count present in aerosols during ultrasonic scaling.
Saini R, 2015 [37]	India	120 patients with chronic periodontitis have been divided arbitrarily into three groups (A, B, and C) of 40 patients each.	ClO2 rinse, Water, CHX gluconate (0.2%)	The outcomes indicated that CFUs in groups A and C were significantly decreased compared to group B.	This study proves that a regular preprocedural mouth rinse could significantly eliminate the majority of aerosols generated by the use of an ultrasonic unit and that ClO2 mouth rinse was found to be statistically equally effective in reducing the aerosol contamination to CHX gluconate (0.2%).

## Conclusions

Mouth rinses can be utilized for several disorders based on the defect seen in the oral cavity. Thus, oral health experts should know several predisposing conditions and etiologic aspects affecting a specific oral defect. The mouth rinse should be used only for a limited period based on the existing lesion. It should always be utilized in addition to mechanical plaque control methods (tooth brushing and flossing). Various dental treatments result in the diffusion of microbes in the dental clinic through aerosol generation. In all the studies included in this review article for periodontal prophylaxis on dental patients, it was discovered that pre-procedural rinsing for 30 seconds to one minute with specific antimicrobial solutions, as opposed to water or no rinsing, significantly reduced aerosol contamination and reduction in the viral load (SARS-CoV-2). There is proof that the antibacterial solution for this, CHX (either 0.12 or 0.2%), works well. Pre-procedural mouth rinses considerably reduce the number of microbes in the dental aerosol.

## References

[1] Kohn WG, Collins AS, Cleveland JL, Harte JA, Eklund KJ, Malvitz DM (2003). Guidelines for infection control in dental health-care settings--2003. MMWR Recomm Rep.

[2] Harrel SK, Molinari J (2004). Aerosols and splatter in dentistry: a brief review of the literature and infection control implications. J Am Dent Assoc.

[3] Zemouri C, de Soet H, Crielaard W, Laheij A (2017). A scoping review on bio-aerosols in healthcare and the dental environment. PLoS One.

[4] Vyas T, Bhatt G, Gaur A, Sharma C, Sharma A, Nagi R (2021). Chemical plaque control - a brief review. J Family Med Prim Care.

[5] Quintas V, Prada-López I, Donos N, Suárez-Quintanilla D, Tomás I (2015). In situ neutralisation of the antibacterial effect of 0.2% chlorhexidine on salivary microbiota: quantification of substantivity. Arch Oral Biol.

[6] Singh N, Charde P, Bhongade ML (2016). Comparative evaluation between honey and chlorhexidine gluconate on the dental plaque levels and gingival health. Adv Dent Oral Health.

[7] Foulkes DM (1973). Some toxicological observations on chlorhexidine. J Periodontal Res Suppl.

[8] Watts A, Addy M (2001). Tooth discolouration and staining: a review of the literature. Br Dent J.

[9] DePaola LG, Overholser CD, Meiller TF, Minah GE, Niehaus C (1989). Chemotherapeutic inhibition of supragingival dental plaque and gingivitis development. J Clin Periodontol.

[10] Mandel ID (1994). Antimicrobial mouthrinses: overview and update. J Am Dent Assoc.

[11] Siegrist BE, Gusberti FA, Brecx MC, Weber HP, Lang NP (1986). Efficacy of supervised rinsing with chlorhexidine digluconate in comparison to phenolic and plant alkaloid compounds. J Periodontal Res.

[12] Overholser CD, Meiller TF, DePaola LG, Minah GE, Niehaus C (1990). Comparative effects of 2 chemotherapeutic mouthrinses on the development of supragingival dental plaque and gingivitis. J Clin Periodontol.

[13] Winn DM, Blot WJ, McLaughlin JK (1991). Mouthwash use and oral conditions in the risk of oral and pharyngeal cancer. Cancer Res.

[14] Beiswanger BB, Mallatt ME, Mau MS, Katz BP (1990). The relative plaque removal effect of a prebrushing mouthrinse. J Am Dent Assoc.

[15] Chung L, Smith SR, Joyston-Bechal S (1992). The effect of using a pre-brushing mouthwash (Plax) on oral hygiene in man. J Clin Periodontol.

[16] Raja M, Saha S, Reddy VK, Mohd S, Kumari M (2013). Mouthwashes-an overview of current knowledge. Int J Oral Health Res Rev.

[17] Palcanis KG, Sarbin AG, Koertge TE, Brooks CN, Gunsolley JC (1990). Longitudinal evaluation of the effect of sanguinarine on plaque and gingivitis. Gen Dent.

[18] Epstein JB, Silverman S Jr, Paggiarino DA (2001). Benzydamine HCl for prophylaxis of radiation-induced oral mucositis: results from a multicenter, randomized, double-blind, placebo-controlled clinical trial. Cancer.

[19] Roberts WR, Addy M (1981). Comparison of the in vivo and in vitro antibacterial properties of antiseptic mouthrinses containing chlorhexidine, alexidine, cetyl pyridinium chloride and hexetidine. Relevance to mode of action. J Clin Periodontol.

[20] Gaffar A, Scherl D, Afflitto J, Coleman EJ (1995). The effect of triclosan on mediators of gingival inflammation. J Clin Periodontol.

[21] Wade AB, Blake GC, Mirza KB (1966). Effectiveness of metronidazole in treating the acute phase of ulcerative gingivitis. Dent Pract Dent Rec.

[22] Adamietz IA, Rahn R, Böttcher HD, Schäfer V, Reimer K, Fleischer W (1998). Prophylaxis with povidone-iodine against induction of oral mucositis by radiochemotherapy. Support Care Cancer.

[23] Tenovuo J (2002). Clinical applications of antimicrobial host proteins lactoperoxidase, lysozyme and lactoferrin in xerostomia: efficacy and safety. Oral Dis.

[24] Marinho VC, Higgins JP, Logan S, Sheiham A (2003). Topical fluoride (toothpastes, mouthrinses, gels or varnishes) for preventing dental caries in children and adolescents. Cochrane Database Syst Rev.

[25] Patel AS, Patel SA, Fulzele PR, Mohod SC, Chandak M, Patel SS (2020). Evaluation of the role of propolis and a new herbal ointment in promoting healing of traumatic oral ulcers: an animal experimental study. Contemp Clin Dent.

[26] Shinada K, Ueno M, Konishi C (2010). Effects of a mouthwash with chlorine dioxide on oral malodor and salivary bacteria: a randomized placebo-controlled 7-day trial. Trials.

[27] Carrouel F, Conte MP, Fisher J, Gonçalves LS, Dussart C, Llodra JC, Bourgeois D (2020). COVID- 19: a recommendation to examine the effect of mouthrinses with β-cyclodextrin combined with citrox in preventing infection and progression. J Clin Med.

[28] Huang YH, Huang JT (2021). Use of chlorhexidine to eradicate oropharyngeal SARS-CoV-2 in COVID-19 patients. J Med Virol.

[29] Costa DD, Brites C, Vaz SN, de Santana DS, Dos Santos JN, Cury PR (2021). Chlorhexidine mouthwash reduces the salivary viral load of SARS-CoV-2: a randomized clinical trial. Oral Dis.

[30] Ferrer MD, Barrueco ÁS, Martinez-Beneyto Y (2021). Clinical evaluation of antiseptic mouth rinses to reduce salivary load of SARS-CoV-2. Sci Rep.

[31] Feres M, Figueiredo LC, Faveri M, Stewart B, de Vizio W (2010). The effectiveness of a preprocedural mouthrinse containing cetylpyridinium chloride in reducing bacteria in the dental office. J Am Dent Assoc.

[32] Reddy S, Prasad MG, Kaul S, Satish K, Kakarala S, Bhowmik N (2012). Efficacy of 0.2% tempered chlorhexidine as a pre-procedural mouth rinse: a clinical study. J Indian Soc Periodontol.

[33] Shetty SK, Sharath K, Shenoy S, Sreekumar C, Shetty RN, Biju T (2013). Compare the effcacy of two commercially available mouthrinses in reducing viable bacterial count in dental aerosol produced during ultrasonic scaling when used as a preprocedural rinse. J Contemp Dent Pract.

[34] Gupta D, Bhaskar DJ, Gupta RK (2014). Effect of Terminalia chebula extract and chlorhexidine on salivary pH and periodontal health: 2 weeks randomized control trial. Phytother Res.

[35] Dawson M, Soro V, Dymock D (2016). Microbiological assessment of aerosol generated during debond of fixed orthodontic appliances. Am J Orthod Dentofacial Orthop.

[36] Retamal-Valdes B, Soares GM, Stewart B (2017). Effectiveness of a pre-procedural mouthwash in reducing bacteria in dental aerosols: randomized clinical trial. Braz Oral Res.

[37] Saini R (2015). Efficacy of preprocedural mouth rinse containing chlorine dioxide in reduction of viable bacterial count in dental aerosols during ultrasonic scaling: a double-blind, placebo-controlled clinical trial. Dent Hypotheses.

